# Photon Counting Computed Tomography for Accurate Cribriform Plate (Lamina Cribrosa) Imaging in Adult Patients

**DOI:** 10.3390/tomography10030031

**Published:** 2024-03-08

**Authors:** Anna Klempka, Eduardo Ackermann, Sven Clausen, Christoph Groden

**Affiliations:** 1 Department of Neuroradiology, University Medical Centre Mannheim, Medical Faculty Mannheim, University of Heidelberg, 68167 Mannheim, Germany; 2 Department of Radiation Oncology, University Medical Centre Mannheim, Medical Faculty Mannheim, University of Heidelberg, 68167 Mannheim, Germany

**Keywords:** photon counting, cribriform plate, skull base, imaging, CT, radiation

## Abstract

Detailed visualization of the cribriform plate is challenging due to its intricate structure. This study investigates how computed tomography (CT) with a novel photon counting (PC) detector enhance cribriform plate visualization compared to traditionally used energy-integrated detectors in patients. A total of 40 patients were included in a retrospective analysis, with half of them undergoing PC CT (Naeotom Alpha Siemens Healthineers, Forchheim, Germany) and the other half undergoing CT scans using an energy-integrated detector (Somatom Sensation 64, Siemens, Forchheim, Germany) in which the cribriform plate was visualized with a temporal bone protocol. Both groups of scans were evaluated for signal-to-noise ratio, radiation dose, the imaging quality of the whole scan overall, and, separately, the cribriform plate and the clarity of volume rendering reconstructions. Two independent observers conducted a qualitative analysis using a Likert scale. The results consistently demonstrated excellent imaging of the cribriform plate with the PC CT scanner, surpassing traditional technology. The visualization provided by PC CT allowed for precise anatomical assessment of the cribriform plate on multiplanar reconstructions and volume rendering imaging with reduced radiation dose (by approximately 50% per slice) and higher signal-to-noise ratio (by approximately 75%). In conclusion, photon-counting technology provides the possibility of better imaging of the cribriform plate in adult patients. This enhanced imaging could be utilized in skull base-associated pathologies, such as cerebrospinal fluid leaks, to visualize them more reliably for precise treatment.

## 1. Introduction

The evolution of the technical capabilities of computed tomography in imaging has always presented a significant challenge to keep up with [1,2]. Following the development of high-quality scanners, there was a noticeable shift towards reducing radiation exposure [3]. Later, achieving detailed visualization became a primary focus. Nowadays, there is access to various state-of-the-art scanners, but the introduction of the latest photon-counting scanners has reinvigorated the quest for further advancements without necessarily increasing radiation, as seen with conventional scanners [4]. This technology holds potential for new and exciting clinical applications [5].

In the first part of the introduction, the technical rationale behind why photon-counting detectors stand out from other types of detectors will be elucidated, while in the second part, the challenges associated with imaging the cribriform plate and its pathologies will be highlighted.

In recent years, the field of medical imaging has witnessed a transformative breakthrough in the form of photon-counting computed tomography (PC CT), which has the potential to address the challenging imaging of small anatomical structures [6,7,8]. The distinctions of this technology lie in the signal processing and offer several advantages in imaging, including higher spatial resolution at the level of 0.2 mm. The unique features of photon- counting (PC) technology have emerged in radiation detectors [9]. Unlike energy-integrated detectors, which rely on scintillator elements and septa, PC detectors directly convert individual photons into electrical signals. This bypasses the need for intermediary steps, such as generating secondary photons, as required by scintillator detectors. Consequently, concerns regarding photon leakage between septa are alleviated, enhancing dose efficiency [10].

PC technology involves coupling semiconductor sensors with a readout circuit, facilitating the direct conversion of X-rays into electrical signals. This streamlined process not only reduces noise but also enhances contrast and spatial resolution without increasing radiation dose. The clinical potential of this fact has already been demonstrated by authors referencing the imaging of the middle ear as well as bone abnormalities [11,12]. This study aims to explore the potential of PC CT using its unique imaging of the anterior part of the skull, particularly the lamina cribrosa.

Detailed imaging of the lamina cribrosa, located in the anterior part of the skull base, poses a significant challenge. This arises from the intricate landscape in which it lies by the presence of low-density substances, namely the air in the nasal cavities, juxtaposed with partial high-density bony structures of the anterior part of the skull base, rendering it a porous and low-density structure [13,14].

Notably, its slender morphology features distinct furrows that provide essential structural support to the olfactory bulb [15]. At its anterior aspect, the cribriform plate interfaces with the frontal bone, forming a robust and compact boundary. It features two small wing-like structures known as alae, which snugly fit into corresponding depressions within the frontal bone [16]. Positioned at the midpoint of the cribriform plate is the crista galli, characterized by its elongated and narrow posterior edge, serving as an attachment point for the falx cerebri. The cribriform plate presents a slender and prominently grooved appearance on both sides of the crista galli [13].

Not only the anatomical structure and variations, but pathologies arising from the lamina cribrosa are also challenging to visualize.

The assessment of the ethmoid roof, described with the Keros classification, is a standard procedure that correlates with the depth of the olfactory fossa [17]. Another classification, known as the Gera classification, assesses the anatomical variation in a horizontal plane of CT reconstruction and evaluates the angle between the lateral lamella of the cribriform plate and the continuation of the horizontal plane passing through the cribriform plate [18,19]. These classifications provide an estimate of risks, such as the risk of iatrogenic cerebrospinal fluid leak, and offer clinicians quantitative descriptions of this variable structure [18,20].

The crucial role of this skull component and its pathological processes takes precedence as this delicate structure serves as the gateway to olfaction. However, it is not merely a passive conduit for olfactory signals; the cribriform plate is also a site where pathology can manifest, with conditions such as cerebrospinal fluid leaks posing diagnostic and therapeutic challenges [21,22,23]. Furthermore, additional conditions such as herniation through the cribriform plate have been documented [24].

The significance of MRI imaging of the skull base is indisputable, as it illustrates the downward sloping of structures and assists in distinguishing between pathologies such as encephalocele or lobe herniation, as well as imaging tumours in the olfactory area. In these processes, visualization of cranial base features (such as pneumatisation, thinning of cortical bone, and extent of destruction) is better achieved with CT, while imaging of the internal soft tissue of the skull is superior with MRI, thus highlighting the complementary nature of these imaging methods.

The aim of this study was to evaluate the efficacy of PC CT as a tool for achieving high-quality cribriform plate imaging in adults. It addressed a critical gap in the current understanding of cribriform plate imaging and contributed to the ongoing advancement of medical imaging techniques, with potential implications for the diagnosis and treatment of various neurological and otorhinolaryngological disorders [25].

## 2. Materials and Methods

Commencing with a retrospective analysis in 2023, the medical records of adult patients who had undergone temporal bone scans at our institution were meticulously examined. Specifically, scans obtained from the Naeotom Alpha Siemens Healthineers, Forchheim, Germany PC CT scanners, as well as the Sensation 64 Siemens, Forchheim, Germany, EID CT scanner, were scrutinized. In total, 40 scans, comprising 20 from each scanner type, were comprehensively reviewed to ensure complete capture of the lamina cribrosa on the CT images.

Imaging of the lamina cribrosa with a temporal protocol is not obligatory, partly due to variations in patient positioning and clinical inquiries. However, CT of the temporal bone is highly precise in imaging, and detailed indications for conducting the examinations for all patients are listed on Table 1. The patient cohort consisted of individuals without known olfactory area symptoms, with demographic details and distribution delineated for both PC CT and EID CT groups. Anatomical abnormalities were meticulously assessed, categorized, and documented in accordance with established classification systems. Furthermore, detailed protocols for CT scans, encompassing imaging parameters and reconstruction techniques, were thoroughly standardized and adhered to for both PC CT and EID CT scans.

### 2.1. Patient Collective

The Shapiro–Wilk test was employed to assess the distribution of the patient collective. The tests yielded a statistic value of 0.9666 and a *p*-value of 0.278, indicating that the age distribution closely approximates a normal distribution, as the *p*-value is significantly above the commonly accepted alpha level of 0.05. This result suggests that the sample comes from a normally distributed population. The statistical analyses pertaining to the age distribution within the studied groups were conducted utilizing Python version 3.10, alongside the scipy.stats module.

The distribution of gender and age in the PC CT group: 13 female and 7 male and 8 female and 12 males in the EID group. The mean age of patients was 52.25 ± 32.20 in the PC CT and 61.25 ± 31.9 in EID CT group.

To assess the cohesion within the group in the face of abnormalities, the type according to the Keros classification, as well as the presence of other anomalies such as dehiscence or aplasia were determined [26]. As illustrated in Table 1, only one anomaly was observed, which was a pneumatized crista galli. The Keros classification results were as follows: in the PC CT group, there were 3 cases of Type 1, 11 cases of Type 2, and 6 cases of Type 3; in the EID CT group, there was 1 case of Type 1, 14 cases of Type 2, and 5 cases of Type 3.

### 2.2. Computed Tomography Scans

Every CT scan of the temporal bone included was standardized. For PC CT: 120 kV, 25 mAs (ref.), ME67, pitch factor 0.85, rotation time 0.5 s. For EID CT: 140 mAs, 120 kV, pitch factor 0.85, rotation time 1.00 s. The quality assurance of the protocols was in accordance with dose and quality assurance guidelines in Germany [27].

### 2.3. Image Preparation—Multiplanar Reconstructions

**Figure 1:**
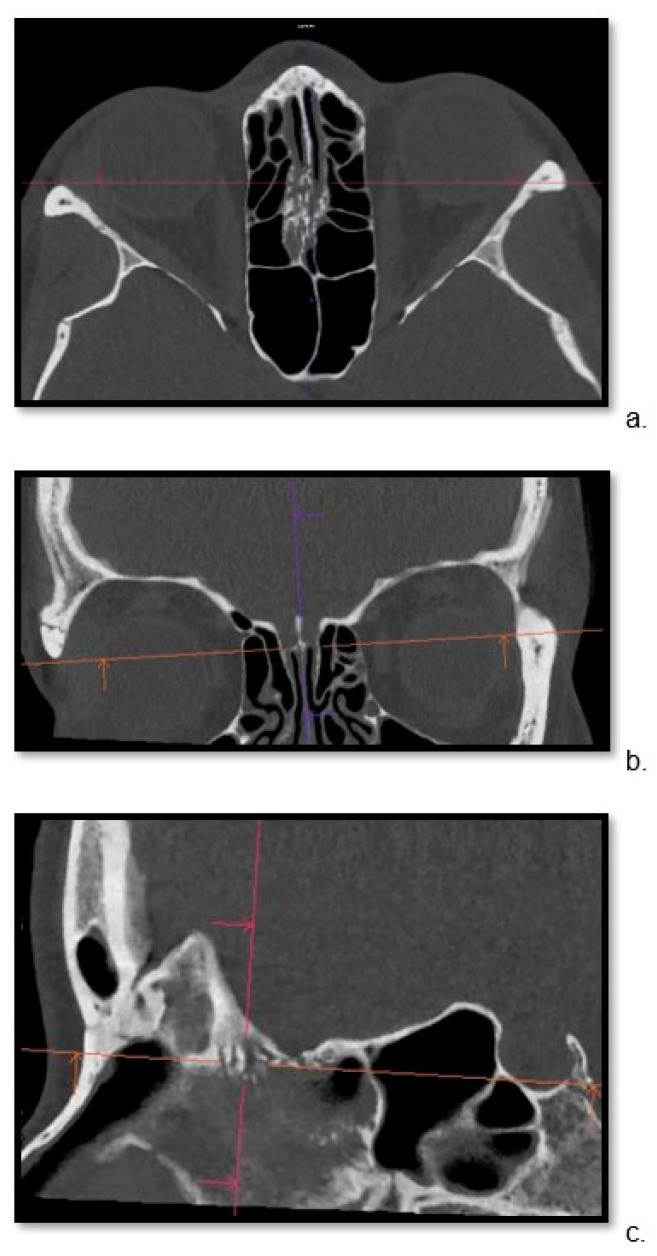
Three-dimensional localization of cribriform plate. Thin-layer imaging of the ethmoidal crest using photon-counting computed tomography. Examination in multiplanar reformation includes (**a**) axial, (**b**) coronal, and (**c**) sagittal views on thin-layer CT.

**Figure 2:**
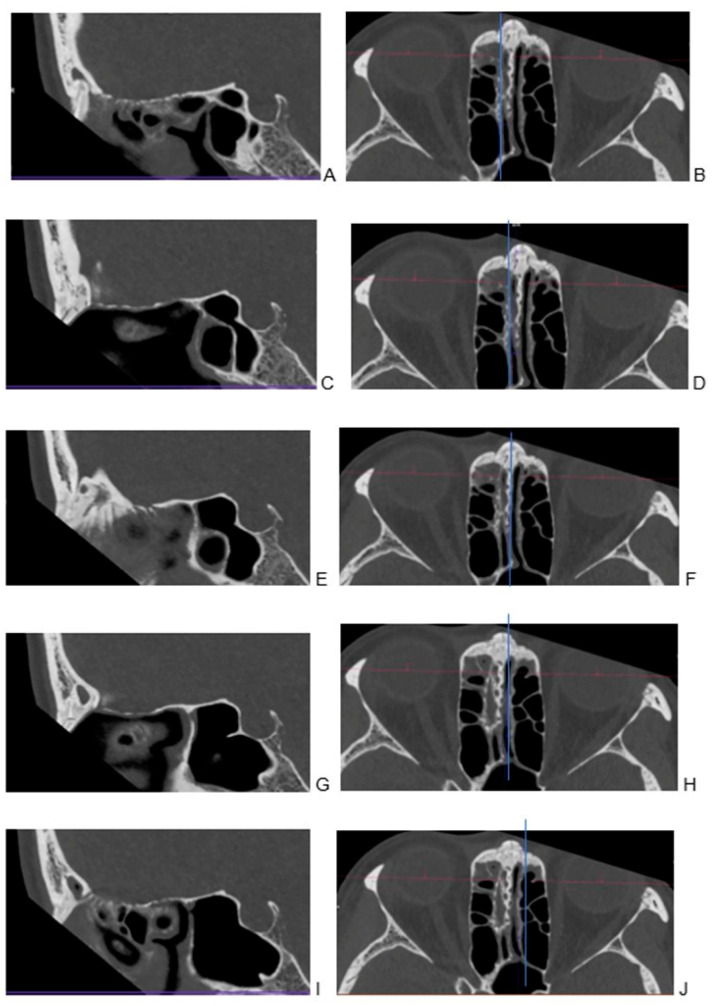
Visualization of cribriform plate in photon-counting computed tomography. Visualization of cribriform plate in photon-counting computed tomography. Examination in multiplanar reformation (**A**–**J**). To facilitate referencing specific layers in sagittal imaging with 0.2 mm, divided into several distinct regions for closer examination: (**A**) the right lateral part of the cribriform plate, showcasing its specific features and characteristics; (**B**) a corresponding transverse view; (**C**) moving towards the right side, the portion situated between the lateral and middle sections of the cribriform plate; (**D**) a corresponding transverse view; (**E**) transitioning to the sagittal plane with a focus on the middle part of the cribriform plate; (**F**) a corresponding transverse view accompanying the sagittal perspective to aid in comprehensive examination; (**G**) on the left side, a space located between the lateral and middle sections; (**H**) a corresponding transverse view provides a complementary angle; (**I**) the left lateral part of the cribriform plate; (**J**) a corresponding transverse view.

**Figure 3:**
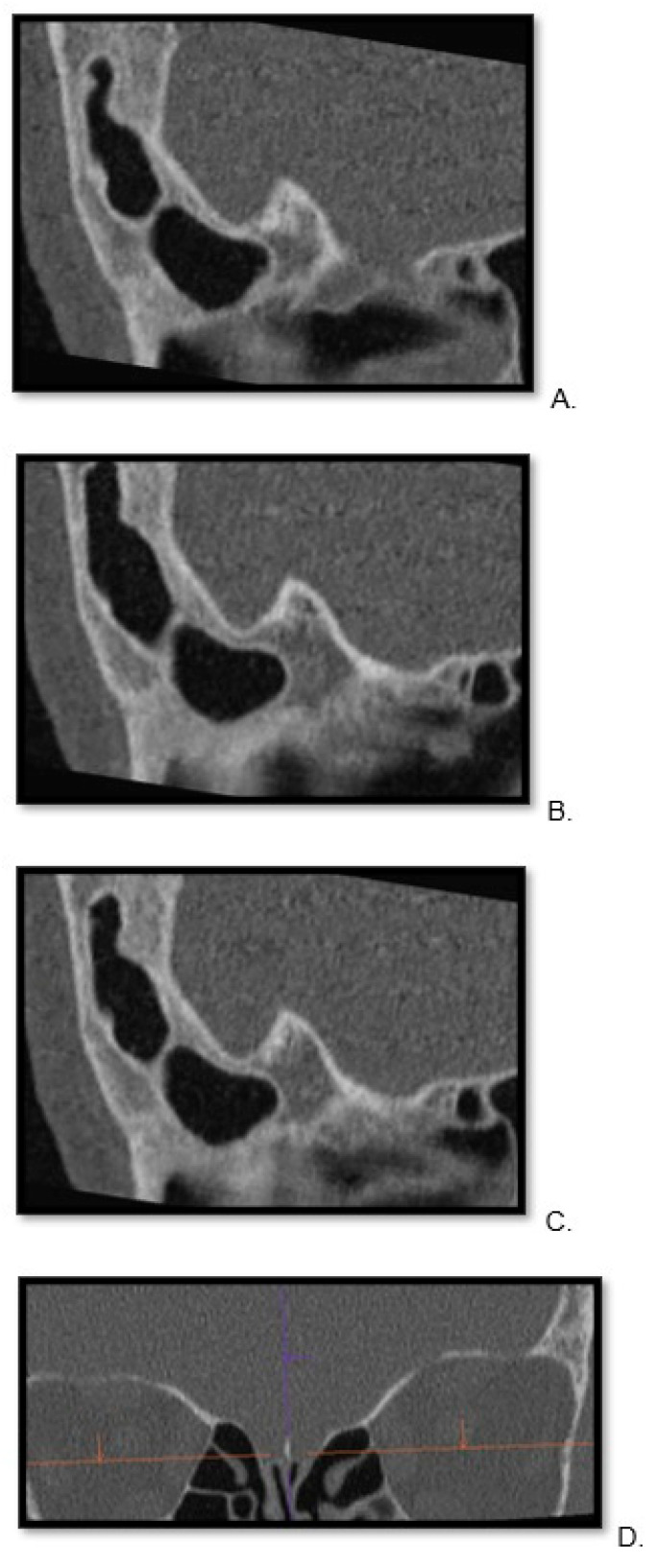
Visualization of cribriform plate in energy-integrated detector computed tomography scan. A lateral visualization of the right to left portions of the cribriform plate in sagittal view (**A**)—right, (**B**)—middle, (**C**)—left side of cribrofirm plate). (**D**) To facilitate referencing specific layers in sagittal imaging with 0.6 mm, we include a coronal view.

For both groups, the thinnest possible reconstruction was prioritized and consistently the hard kernel was utilized. The kernel settings were in line with the standard bone reconstruction settings used in our institution for each scanner. For PC CT, Hr84 was used with WW/WL settings of 700/3000 and a minimum slice thickness of 0.2 mm, while for EID CT, the WW/WL settings were 700/4000 U90u with a minimum slice thickness of 0.6 mm. For each patient, the Siemens’ Syngo.via Client 8.3 (Siemens Healthcare) software was used to localize the cribriform plate in three planes (Figure 1) and the sagittal view through the ala cribriform plate in the thinnest possible layer was used (Figure 2 and Figure 3). The identification of the crista galli was crucial for the preparation of sagittal views.

### 2.4. Image Preparation—Volume Rendering Technique

**Figure 4:**
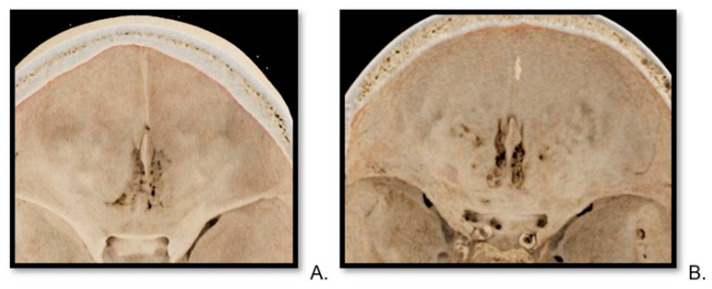
Reconstructions of cribriform plate in photon-counting computed tomography. A top-view VRT (volume rendering technique) of cribriform plate, reconstruction from photon-counting computed tomography scan of a 30-year-old female (**A**) and a 75-year-old male (**B**).

**Figure 5:**
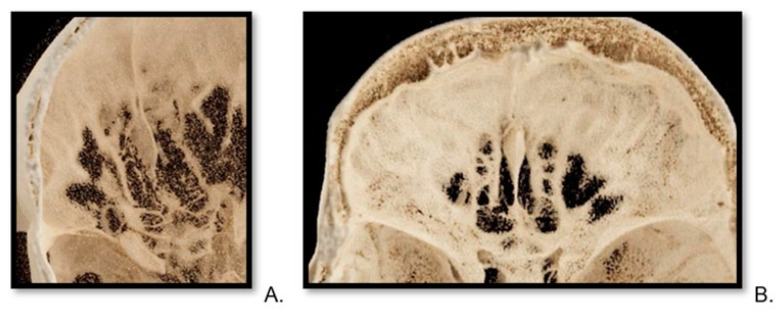
Reconstructions of cribriform plate in energy-integrated detector computed tomography scan. A top-view VRT (volume rendering technique) image of a cribriform plate of a 33-year-old male patient (**A**) and a 61-year-old female (**B**).

VRT (volume rendering technique) reconstructions were generated for each patient by an experienced radiologist with 10 years of clinical expertise (Figure 4 and Figure 5). For VRT, the thinnest data stack was also used with the software Syngo.via Client 8.3 by Siemens Healthcare, always utilizing the same graphic pattern from cinematic rendering [28]. This reconstruction allowed for a free insight into the frontal skull from the inside from a cranial projection. The possibility of modifying the angulation of the reconstruction or magnifying the picture, for example, to see the lateral lamina cribrosa openings, was available.

### 2.5. Evaluation of Quantitative Parameters

Signal-to-Noise Ratio (SNR): SNR was calculated by measuring the mean Hounsfield unit (HU) value within the air in the ethmoidal cells on the left with 20 mm^2^ ROI size and dividing it by the standard deviation of the background air [29]. SNR measurements were obtained for both PC CT and EID CT images. Simplified analysis of comparison of radiation exposure was conducted. Due to variations in scan length, CTDI (computed tomography dose index) per scan layer or per slice was compared.

### 2.6. Evaluation of Qualitative Parameters

Two observers, each with approx. 10 years of clinical radiology experience, independently assessed the anonymized images for each patient with a 5-point Likert scale (1 = Poor, 5 = Excellent) for image quality of CT scan, visualization of the cribriform plate on CT scan, and quality of the VRT reconstructions in context of the lamina cribrosa [30]. Two experienced radiologists were able to independently perform high-quality judgments of cribriform plate imaging. This aspect of the study design was inspired by literature research in the topic area [16].

### 2.7. Statistical Analysis

The differentiation in ratings between PC CT and EID CT, as well as inter-rater agreement, were analysed. Python version 3.10 together with the SciPy library was utilized for the statistical analysis. The distribution of the data was examined using the Shapiro–Wilk test [31].

### 2.8. AI-Assisted Tools

The grammar-checking procedure for this manuscript was performed using ChatGPT 3.5, which is a language model made by OpenAI to develop artificial intelligence. While ChatGPT 3.5 was the software used to check the grammar of this manuscript, the final proofreading and any changes made were performed by the authors to retain the level of scientific accuracy and clarity. The Introduction, Results, Discussion and Methods sections presented in the submitted manuscript were creatively crafted by the authors.

## 3. Results

### 3.1. Evaluation of Quantitative Parameters

#### 3.1.1. Signal-to-Noise Ratio

This study aimed to achieve a significantly improved SNR in PC CT. The mean SNR for PC CT was 31.81 ± 9.61, while for EID CT it was 7.88 ± 2.07 (Table 2).

#### 3.1.2. Radiation Dose

The mean CTDI for PC CT was 15.42 mGy, whereas in the EID CT group, it was 32.32 mGy. This analysis provides insights into the radiation doses used in the two groups by showing approximately 50% less radiation dose per slice in PC CT (Table 2).

### 3.2. Evaluation of Qualitative Parameters

#### 3.2.1. Image Quality of CT Scan

The image quality of PC CT had a mean of 4.5, whereas EID CT scored 2.6 in a Likert scale (Table 3). The Mann–Whitney U test for the quality assessments was found to be 1576.5, with an associated *p*-value of *p* = 1.885 × 10^−15^. This demonstrates that there is a statistically significant difference in the quality of the conducted tests between PC CT and EID CT.

#### 3.2.2. Image Quality of Cribriform Plate Imaging on CT Scan

**Figure 6:**
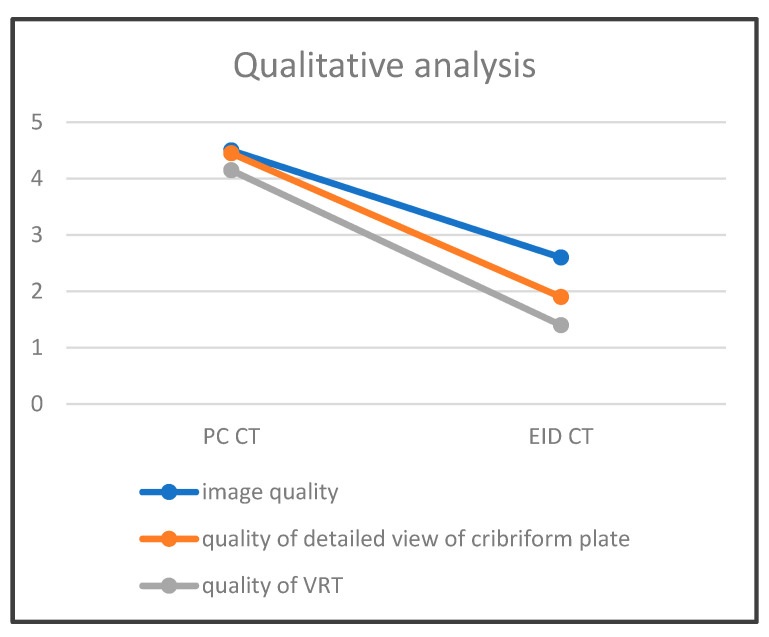
Schematic representation comparing image quality assessed by two observers, considering the average values. Here, the illustration demonstrates a better agreement in high-quality ratings with photon-counting CT (PC CT) than energy-integrated detector CT (EID CT) for image quality, quality of detailed view of cribriform plate, as well as VRT (volume rendering technique).

A detailed view of the cribriform plate scored 4.45 for PC CT and 1.9 for EID CT on a Likert scale (Figure 6, Table 3). Furthermore, the Mann–Whitney U test for the assessment of the cribriform plate on CT was 1553.5, with a *p*-value of *p* = 7.341 × 10^−14^. Again, this highly significant *p*-value demonstrated a clear distinction in ratings between the two methods.

#### 3.2.3. Quality of VRT Reconstructions

The detailed view of the cribriform plate on VRT in PC CT was rated 4.15 compared to 1.4 for EID CT on a Likert scale. The Mann–Whitney U test for the quality of VRT reconstructions was slightly different at 1548.5, yet the *p*-value of *p* = 1.243 × 10^−13^ further indicated statistically significant differences in the quality of VRT reconstructions quality.

#### 3.2.4. Interobserver Agreement

The calculated overall agreement of observers (Cohen’s Kappa) across all was 0.27 for PC CT and 0.26 for EID. Values of Cohen’s Kappa in the range > 0.20 to 0.40 indicate moderate agreement. Therefore, values of Cohen’s Kappa of 0.27 and 0.26 can be interpreted as reflecting moderate agreement between observers for each group. The observer judgment is fully disclosed in Table 3. Please note that in many cases with PC CT, the disagreement was at the levels of 4 (very good) and 5 (excellent), while a value of 3 was assigned only three times in the PC CT group, which contrasts with the judgment in the EID group with lower scores.

## 4. Discussion

The enhanced imaging technique presented in this study could prove to be crucial in investigating numerous pathologies within the olfactory cleft area and the broader anterior skull base [32]. The actual impact of imaging on clinical decision-making still needs to be validated, but the ability to improve image quality without additional radiation exposure, and even reducing radiation, represents a significant breakthrough in detector development. The enhanced precision achieved through multiplanar reconstruction and VRT reconstruction techniques in this study demonstrates substantial potential for achieving precise diagnostic accuracy and optimizing surgical access [33,34]. Furthermore, this advancement may influence the educational processes of future medical professionals [35].

Additionally, the enhanced precision of anatomical details may facilitate the creation of 3D models or printed models for educational purposes and preoperative planning in the anterior skull region [36,37]. Offering clearer and more detailed images may contribute to more effective treatment strategies and improved patient outcomes. The integration of advanced imaging technology into clinical practice represents a significant advancement in modern medicine, with far-reaching implications for both diagnosis and treatment.

One limitation of this study is its simplistic approach, as it only included patients without any known symptoms of the olfactory area. This may limit the generalizability of the findings to a broader population with varied clinical presentations. Additionally, this study did not compare or visualize pathologies of the cribriform plate area, which could offer valuable insights into the diagnostic utility of this imaging technique, as a future project. However, despite these limitations, this research has notable strengths. The retrospective nature of the data collection allowed for the correlation of findings among patient groups. This approach enabled the identification of trends in statistical corelating groups and covers some of the nuances associated with age and gender, aligning with larger cohort studies [38,39].

This study can bring insight into possibilities of recognising pathologies with improved imaging, including phenomena such as olfactory cleft dilatation, which involves the widening or enlargement of the olfactory cleft. Another significant pathology is cerebrospinal fluid leak. PC CT scanning may provide valuable insights into the osseous borders of cerebrospinal fluid drainage in the anterior skull base [40]. The potential for a high signal-to-noise ratio might aid in the identification of cribriform dural arteriovenous fistulas and further enhance the study of radiological anatomy [41,42,43,44]. The results of our study may also support the management of fractures involving the naso-ethmoid or naso-orbital-ethmoid regions, as highlighted by Rosenberger et al. (2013) and Wei et al. (2015) [45,46].

Some studies discussing novel imaging techniques centre on research conducted with small cohorts and samples from cadavers, as demonstrated by Ganei et al. [16]. However, these findings may be already deemed outdated. This research was conducted on live patients and yielded superior results of PC CT. The methodologies outlined in these studies, particularly those concerning bone density in the olfactory area, warrant replication in living patients to validate their clinical significance.

The discussion on radiation exposure in this study underscores the importance of balancing diagnostic precision with patient safety, particularly in the context of CT. This study demonstrates that PC CT improved imaging capabilities while concurrently minimizing radiation doses [47,48]. This research not only seeks to refine comprehension of the cribriform plate’s anatomy and its role in olfaction but also holds promise for diagnostic and therapeutic applications.

## 5. Conclusions

In summary, this study underscores the superior imaging quality attained with the PC CT scanner, as evidenced by both qualitative and quantitative assessments. The enhanced visualization of the cribriform plate without the need for additional radiation exposure marks a notable advancement in diagnostic precision and therapeutic strategies. These findings underscore the ongoing evolution and enhancement of imaging modalities within the realm of computed tomography.

## Figures and Tables

**Table 1:** Indications to conduct temporal bone CT. The table describes two patient groups in the study, indicating the need for a temporal bone CT scan, along with the age and gender of each patient. Additionally, the Keros classification is included. Patient anomalies are marked with the prefix * and described below.

Nr.	Age/Gender	Indication to Conduct Temporal Bone CT	Keros Classification
1 ^1^	21/female	Post-cochlear implantation follow-up	1
2 ^1^	26/female	Post-cholesteatoma with progressive hearing loss	3
3 ^1^	30/female	Tympanic membrane perforation	3 *
4 ^1^	43/male	Bilateral external auditory canal atresia	2
5 ^1^	53/female	Semicircular canal dehiscence	2
6 ^1^	54/male	Sudden hearing loss	3
7 ^1^	66/male	Before left-side cochlear implantation	2
8 ^1^	66/female	Cholesteatoma surgery, suspected recurrence	2
9 ^1^	67/male	Before a cochlear implantation	2
10 ^1^	75/male	Before a cochlear implantation	2
11 ^1^	67/female	Cholesteatoma, otosklerose	3
12 ^1^	27/female	Cholesteatoma and before cochlear implantation	3
13 ^1^	23/male	Otorrhoea right, after tympanoplastic	2
14 ^1^	39/female	Surditas right	2
15 ^1^	67/female	Tinnitus	1
16 ^1^	48/male	Before cochlear implantation	2
17 ^1^	64/female	Before cochlear implantation	2
18 ^1^	56/female	Postoperative control after petrosal meningioma	3
19 ^1^	70/female	Before cochlear implantation	2
20 ^1^	57/female	Before cochlear implantation	1
21 ^2^	29/female	Suspicion of cholesteatoma	3
22 ^2^	33/male	Status post-mastoidectomy, suspected complications	2
23 ^2^	53/male	Before a cochlear implantation	2
24 ^2^	60/female	Before a cochlear implantation	2
25 ^2^	61/female	Before a cochlear implantation	2
26 ^2^	62/male	Bone erosion by squamous cell carcinoma Infiltration	3
27 ^2^	63/female	Before a cochlear implantation	2
28 ^2^	65/male	Before a cochlear implantation	2
29 ^2^	74/male	Revision of a cochlear implantation	3
30 ^2^	90/female	Malignant external otitis	2
31 ^2^	43/female	Before a cochlear implantation	2
32 ^2^	57/female	Cholesteatoma left	2
33 ^2^	59/female	Cholesteatoma left	2
34 ^2^	73/female	Glomus tympanicum right	2
35 ^2^	46/male	Suspicion of otitis externa maligna left	3
36 ^2^	52/male	Before a cochlear implantation	2
37 ^2^	82/female	Surtitas bds before a cochlear implantation	1
38 ^2^	75/female	Before a cochlear implantation	2
39 ^2^	41/male	Suspicion of cholesteatoma	3
40 ^2^	34/female	Suspicion of cholesteatoma	2

^1^ Photon counting computed tomography. ^2^ Energy-integrated detector computed tomography. * Pneumatized crista galli.

**Table 2:** Quantitative analysis. The table presents an analysis of the signal-to-noise ratio (SNR) and CTDI (computed tomography dose index) in mGy for each patient, with patient numbers matching those in Table 1 and Table 3.

Nr.	CTDTI mGy	SNR
1 ^1^	15.0	51.1
2 ^1^	15.2	21.3
3 ^1^	13.9	30.8
4 ^1^	17.8	30.7
5 ^1^	13.7	35.4
6 ^1^	14.5	34.2
7 ^1^	16.6	32.4
8 ^1^	19.4	37.4
9 ^1^	16.2	33.8
10 ^1^	16.6	34.8
11 ^1^	14.6	38.8
12 ^1^	15.7	48.4
13 ^1^	16.1	22.7
14 ^1^	12.3	24.0
15 ^1^	14.0	35.9
16 ^1^	16.1	32.5
17 ^1^	16.2	23.3
18 ^1^	14.4	25.8
19 ^1^	15.8	6.4
20 ^1^	14.8	24.4
21 ^2^	32.32	5.4
22 ^2^	32.32	8.7
23 ^2^	32.32	7.2
24 ^2^	32.32	12.2
25 ^2^	32.32	8.4
26 ^2^	32.32	8.9
27 ^2^	32.32	9.8
28 ^2^	32.32	9.3
29 ^2^	32.32	7.9
30 ^2^	32.32	8.9
31 ^2^	32.32	4.9
32 ^2^	32.32	8.2
33 ^2^	32.32	8.0
34 ^2^	32.32	8.5
35 ^2^	32.32	9.8
36 ^2^	32.32	7.6
37 ^2^	32.32	10.3
38 ^2^	32.32	9.6
39 ^2^	32.32	5.0
40 ^2^	32.32	5.4

^1^ Photon counting computed tomography. ^2^ Energy-integrated detector computed tomography.

**Table 3:** Qualitative analysis. Patient descriptions were rated on a Likert scale from 1 to 5, where 5 signifies the highest quality. The first number represents the judgment of the first observer, while the number following the ‘/’ represents the assessment by the second observer for each patient, with patient numbers corresponding to those in Table 1 and Table 2.

Nr.	Image Quality of Imaging of CT Scan	Image Quality of Cribriform Plate on CT Scan	Quality of VRT Reconstructions
1 ^1^	5/4	5/4	5/5
2 ^1^	5/5	5/5	5/5
3 ^1^	5/4	5/4	5/5
4 ^1^	5/5	5/4	5/5
5 ^1^	5/5	5/5	5/4
6 ^1^	5/5	5/4	5/5
7 ^1^	5/5	5/5	4/4
8 ^1^	5/5	5/5	5/5
9 ^1^	5/5	5/5	5/3
10 ^1^	5/5	5/4	5/5
11 ^1^	5/5	5/5	5/4
12 ^1^	5/5	5/5	5/5
13 ^1^	5/5	5/4	5/4
14 ^1^	5/5	4/5	5/5
15 ^1^	5/5	4/4	4/3
16 ^1^	5/4	4/3	4/4
17 ^1^	5/5	5/4	3/3
18 ^1^	5/4	5/3	3/2
19 ^1^	4/3	3/2	2/2
20 ^1^	5/5	5/4	4/4
21 ^2^	4/3	4/2	1/1
22 ^2^	4/3	4/3	2/2
23 ^2^	2/1	2/1	3/1
24 ^2^	3/2	3/1	3/2
25 ^2^	3/2	3/1	2/1
26 ^2^	3/1	3/1	2/1
27 ^2^	2/2	2/2	4/3
28 ^2^	3/3	3/2	2/2
29 ^2^	2/2	2/1	2/1
30 ^2^	2/3	2/1	3/1
31 ^2^	3/3	2/2	1/1
32 ^2^	3/3	2/1	1/1
33 ^2^	3/3	2/2	1/1
34 ^2^	3/4	3/2	2/1
35 ^2^	3/3	2/2	2/1
36 ^2^	3/3	2/1	1/1
37 ^2^	3/3	2/2	1/2
38 ^2^	2/3	2/1	1/1
39 ^2^	3/3	2/2	1/1
40 ^2^	3/3	2/2	1/1

^1^ Photon counting computed tomography. ^2^ Energy-integrated detector computed tomography.

## Data Availability

The data presented in this study are available on reasonable request from the corresponding author.
